# Trans-Oral Robotic Surgery (TORS) and Postoperative Hemorrhage: An Analysis of Risk Factors

**DOI:** 10.3390/jpm15050201

**Published:** 2025-05-16

**Authors:** Andrea Migliorelli, Elia Biancoli, Marianna Manuelli, Alberto Caranti, Andrea Ciorba, Chiara Bianchini, Giuseppe Meccariello, Claudio Vicini

**Affiliations:** 1 ENT & Audiology Unit, Department of Neurosciences, University Hospital of Ferrara, 44100 Ferrara, Italy; 2 Otolaryngology Unit, Department of Neurosciences (DNS), University of Padova, 35128 Padua, Italy; 3 Gruppo Otorinolaringoiatrico della Romagna, GVM Care & Research, 48018 Faenza, Italy; dott.albertocaranti@gmail.com (A.C.);; 4 ENT Department, Ospedale Morgagni Pierantoni, AUSL Romagna, 47121 Forlì, Italy

**Keywords:** trans-oral robotic surgery, TORS, postoperative hemorrhage, risk factors, OSA, OPSCC

## Abstract

**Background/Objectives:** Postoperative hemorrhage is the most common complication after Trans-Oral Robotic Surgery (TORS) described in the literature. The aim of this study is to assess the presence of any risk factors that may impact postoperative bleeding. **Methods**: This was a retrospective study based on the analysis of patient data. Patients undergoing TORS procedures at the ENT Unit of Forlì Hospital from 2008 to 2022 for OSA (obstructive sleep apnea) or oncological disease and with a minimum follow-up of 30 days were included. The comorbidities analyzed were perioperative anticoagulant/antiplatelet therapy and clinicopathological features concerning the pathology. Total bleeding and severe bleeding (which required management in the operating room) were included. **Results**: A total of 414 patients (106 oncological TORS and 308 OSA TORS patients) were included. Post-TORS bleeding occurred in 47 cases (11.3%) and severe bleeding in 18 cases (4.3%). The pathology (oncology vs. OSA) treated with TORS did not represent a risk factor (*p* = 0.466). Antiplatelet intake represented an important risk factor (*p* = 0.002). Postoperative hemorrhage for oncological TORS occurred in 11.3% patients; of these, 6.6% had severe bleeding. Artery ligation during neck dissection prevented the risk of severe bleeding (*p* < 0.001). In TORS for OSA, postoperative hemorrhage was found in 11.4% cases, of which 3.6% were major bleeding. Neither the degree of OSA nor the association with other concurrent procedures were risk factors for postoperative bleeding in this study. **Conclusions**: Patients taking perioperative antiplatelet therapy have an almost 5-fold increased risk of developing postoperative bleeding. The pathology (oncology vs. OSA) does not influence the risk of bleeding. Prophylactic arterial ligation during neck dissection significantly decreases the risk of severe bleeding.

## 1. Introduction

In recent decades, robotic surgery has become a widely implemented technique in multiple areas, including the head and neck region. Compared to conventional surgery, Trans-Oral Robotic Surgery (TORS) offers a precise, safe, and effective approach to body areas with challenging accesses [1]. TORS was initially employed in the surgical management of tumors in the oropharyngeal region. Following its approval by the FDA in 2009 [2], there has been a rapid increase in clinical and scientific interest in TORS, including in the treatment of benign pathologies. The introduction of TORS has innovated the surgical treatment of obstructive sleep apnea (OSA) by allowing for safer and more accurate management of the base of the tongue [3].

Despite the favorable safety profile of TORS, as with all surgical procedures, some complications can occur in the postoperative period. The most frequently documented complications include hemorrhage, dental lesions, nasogastric tube dependency, dysphagia, lingual and/or hypoglossal nerve injuries, fistulas, and upper airway obstruction [3,4,5,6,7]. Postoperative hemorrhage remains the most common complication to date, and, in rare cases, it can be severe and even fatal [5,8,9,10,11].

The primary aim of this retrospective study was to assess the presence of any risk factor that may increase the risk of postoperative bleeding in patients treated by TORS, enhancing the precision of the surgical procedure for both oncological pathology and OSA. Furthermore, in the oncology subgroup, the role of arterial ligation (AL) (external carotid artery/lingual artery) in preventing postoperative bleeding was evaluated.

## 2. Materials and Methods

This was a retrospective study. The medical records of all patients undergoing TORS for oncological reasons or for OSA at the ENT Department of the Morgagni-Pierantoni Hospital in Forlì between 1 January 2008 and 31 December 2022 were revised. The inclusion criteria were (i) patients undergoing TORS during the specified period; (ii) patients with oncological pathology or OSA; (iii) patients with a minimum follow-up duration of 30 days. The exclusion criteria were (i) patients with a history of other surgeries in the cervico-facial region; (ii) patients undergoing TORS for pathologies other than oncology and OSA; (iii) patients with a follow-up of less than 30 days.

The clinicopathological features of all patients included the cause leading to TORS (oncological vs. OSA) treatment, gender, age at the time of surgery, smoking, alcohol consumption, and body mass index (BMI). The latter was categorized into three groups: a normal-weight group (BMI < 25), an overweight group (BMI > 25 and <30), and an obese group (BMI > 30). The presence or absence of any pathologies at the time of the procedure was also assessed; these included hypertension, diabetes mellitus, cardiac pathologies (e.g., atrial fibrillation and previous myocardial infarctions), pathologies leading to impaired coagulation, pulmonary pathologies (e.g., asthma, chronic obstructive pulmonary disease), nephrological diseases (e.g., chronic kidney disease), liver diseases (e.g., cirrhosis), rheumatological diseases (e.g., vasculopathies, Sjogren’s disease), thyroid diseases (e.g., hypothyroidism), gastrointestinal diseases (e.g., Crohn’s disease, ulcerative rectocolitis), the presence of gastroesophageal reflux, congenital diseases (e.g., Down’s disease), and the presence of previous tumors.

Additionally, preoperative/intraoperative parameters were assessed, including the Charlson Comorbidity Index [12], the International Normalized Ratio (INR) (considered pathological if > than 1.2), activated partial thromboplastin time (APTT) (considered pathological if > than 1.2), platelet value (considered normal between 150 and 450 × 10^3^/mL), antiplatelet (cardioaspirin, clopidogrel) and/or anticoagulant (warfarin, direct oral anticoagulants, low-molecular-weight heparins) intake at the time of surgery, the STOP BANG (test assessing the likelihood of the patient having OSA) [13], the El-Ganzouri test (a test to assess the likelihood of difficult orotracheal intubation) [14], the American Society of Anesthesiology (ASA) classification [15], whether intraoperative tracheotomy was performed, and decannulation time.

Finally, the occurrence of any postoperative bleeding (within 30 days after surgery) was assessed. In case bleeding was registered, the extent of the bleeding was evaluated also.

The definition of major (severe) bleeding as used in this study was based on the requirement for surgical revision in the operating room. Consequently, this study includes both total bleeding (major and non-major bleeding) and isolated major bleeding. Furthermore, the postoperative day on which bleeding occurred was assessed, as well as whether this required an emergency tracheotomy (in case it had not been performed during the primary surgery).

For patients with an oncological diagnosis, this study focused exclusively on squamous-cell carcinoma, thereby excluding all other histotypes.

The present study was conducted in accordance with the Declaration of Helsinki (2008). It was conducted retrospectively through a systematic review of hospital case histories and therefore did not affect patient care in any way, as it was limited to the creation of a database and its evaluation.

### 2.1. Subgroup-Specific Evaluations

For each oncological patient, the following parameters were analyzed: anatomical site, American Joint Committee on Cancer (AJCC) global stage (8th edition) [16], any prior radiation therapy, surgical approach with/without neck dissection, flap reconstruction, presence of extra-nodal extension, grading, vascular infiltration, Human Papillomavirus (HPV) and p16 status, margin status, postoperative complications within 30 days, potential postoperative radiotherapy, local recurrence, regional recurrence, and occurrence of distant metastases.

Furthermore, the disease-free time interval, overall survival, and follow-up period were also evaluated.

Finally, for patients treated by neck dissection, we evaluated whether arterial ligation (AL) had been performed during the procedure.

For patients undergoing TORS for OSA, we also evaluated the severity of OSA, the preoperative Apnea Hypopnea Index (AHI), the Lowest Oxygen Saturation (LOS), and the presence of any multilevel treatment that might include nasal surgery (e.g., septoplasty) and/or pharyngoplasty.

### 2.2. Statistical Analysis

A descriptive analysis of all variables with their frequencies was performed. The numerical data were expressed as absolute values, percentages, mean ± standard deviation, or median (range).

The statistical significance of any differences between groups with regard to categorical variables was determined by means of the chi-square test or, in the case of 2 × 2 tables and expected frequencies <5%, the Fisher exact test.

Student’s *t*-test was applied for continuous data when the variable data demonstrated a normal distribution.

A multivariate logistic regression analysis was performed to calculate the independent risk of post-surgical bleeding, expressed as an Odds Ratio (OR). Probability values lower than 0.05 were considered statistically significant. All analyses were performed using Statistical Package for the Social Sciences (SPSS) 29.0.1 software (IBM Corp., Armonk, NY, USA).

## 3. Results

A total of 414 patients treated by TORS were included in the study. Table 1 shows the clinical features of the cohort; 106 (25.5%) patients were treated by TORS for oncological pathology, while 308 (74.5%) were treated for OSA. The sample consisted of 323 (78%) males and 91 (22%) females, with a mean age at the time of surgery of 54.09 years (SD ± 12.45); 67.3% of patients were non-smokers, while approximately 55% of patients consumed alcohol daily. Most of the treated patients were overweight (51.5%) and 27.6% were classified as obese according to their BMI.

In the present study, 21.5% of patients were found to be taking anticoagulant medication at the time of surgery according to the guidelines; 4.5% of patients were taking antiplatelet medication at the time of surgery (the treatment was not suspended, as per the guidelines). The INR, APTT, and platelet count were found to be within normal limits in 94.8%, 95%, and 94% of cases, respectively.

Postoperative bleeding occurred in 47 cases and occurred on average 4.51 (SD ± 3.6) days after surgery. Of these cases, eighteen (4.3%) patients required urgent access to the operating room, and in two cases (both following TORS for OSA), an emergent tracheotomy was performed.

Statistical analysis demonstrated a significant correlation between liver disease and bleeding (*p* = 0.041) (Table 2). In addition, the pathology (oncology vs. OSAS) that required TORS did not represent a statistically significant risk factor for bleeding (*p* = 0.466) (Table 3).

Among the factors analyzed, the presence of coagulopathy was identified as a risk factor for post-surgical bleeding (*p* = 0.049). Antiplatelet intake was found to be a statistically significant risk factor (*p* = 0.002), while the intake of anticoagulants did not represent an increased risk of bleeding (*p* = 0.286).

None of the other factors analyzed and summarized in Table 3 was associated with an increased risk of bleeding.

However, an analysis of the 18 cases of severe bleeding (Table 4) revealed that none of the factors analyzed was associated with an elevated risk.

Furthermore, patients who underwent TORS for OSA were younger (*p* < 0.001) and were decannulated earlier (*p* < 0.001) when compared to those treated for oncological disease. Sex was not a risk factor (*p* = 0.191). Intraoperative tracheotomy was performed more frequently in patients with OSAS than in those with cancer (*p* < 0.001) (Table 5).

### 3.1. Oncological Sample

Of the 414 patients included in this study, 106 (25.5%) underwent TORS as part of their oncological treatment (Table 6). Of these, 79 (74.5%) were males and 24 (25.5%) were females, with a mean age of 65 (SD ± 9.6) years. The most prevalent site was the oropharynx, with palatine tonsils being the most frequently involved subsite (42.6%), followed by the base of the tongue (39.7%). In 86 cases (81.1%), neck dissection was performed (and it was a selective procedure for 80 patients). Intraoperative tracheotomy was performed in 56 (52.8%) patients, with an average decannulation time of 9.9 (SD ± 17) days. During neck dissection, AL was performed in 24 cases (22.6%), and no complications were reported. Local flaps were performed in five patients with T2 classification, while anterolateral free thigh (ALT) flaps were performed in six patients with T3 classification. Facial artery myo-mucosal (FAMM) flaps and two buccinator-based myo-mucosal (BMM) flaps were utilized to cover extensive carotid artery exposure in three distinct tonsillar cancer patients (T2). In one case of oropharyngeal cancer involving part of the soft palate and the anterior tonsillar pillar (T2), a temporal myofascial flap (TMF) was adopted to restore competent velopharyngeal sphincter function and a tight seal between the pharynx and the neck. An infrahyoid flap was used to reconstruct a posterior defect at the tongue base (T2). Postoperative bleeding occurred in 12 (11.3%) patients; of these, 7 (6.6%) required a second procedure to stop the bleeding. The bleeding occurred, on average, 4.09 (SD ± 4) days after primary surgery.

According to Table 7, there was no statistically significant correlation between tumor-related factors and the risk of bleeding. However, patients undergoing AL had a lower probability of bleeding (*p* < 0.001) (Table 8). Furthermore, no statistically significant association was observed between preoperative radiotherapy and bleeding risk.

### 3.2. OSA Sample

Of the 308 patients treated for OSA, 244 (79.2%) were males and 64 (20.8%) were females, with a mean age of 50.34 (SD ± 11.02) years (Table 9). The patients had a mean preoperative AHI of 39.68 (SD ± 20.8) and a mean LOS of 77.3 (SD ± 9.9). OSA was classified as mild in 10.9% of cases, moderate in 27.4% of cases, and severe in 61.7% of cases. In 233 cases (75.6%), TORS was part of a multilevel procedure associated with septoturbinoplasty and/or pharyngoplasty. Intraoperative tracheotomy was performed in 268 cases (87%), with a mean decannulation time of 4.2 days (SD ± 2.08).

Postoperative bleeding was observed in 35 cases (11.4%); 11 (3.6%) were classified as major bleeding and 2 cases required emergent tracheotomy. Postoperative bleeding occurred, on average, 4.77 (SD ± 3.7) days after surgery. There was no statistically significant correlation between postoperative bleeding and the degree of OSA or its association with other concurrent procedures (Table 10).

## 4. Discussion

Nowadays, TORS is considered a prevalent and well-known technique for the management of malignant and benign head and neck pathologies [2,7,17,18,19]. The present study reports on a comprehensive case series of 414 patients, revealing that the majority were affected by OSA (74.5%) and were males (78%) with a mean age of 54.09 years (SD ±12.45); those treated for oncological pathology had a higher mean age (65 ± 9.6 vs. 50.34 ± 11.02). To the best of our knowledge, no other studies have directly compared the two most common indications for TORS (OSA vs. oncology). It is also important to highlight that TORS treatment allows the surgical approach to be personalized in terms of a more tailored and precise treatment.

Postoperative hemorrhage has been reported to be the most prevalent complication of TORS procedures. Chia et al. [5] and Chen et al. [11] reported rare cases of death following severe hemorrhage in their studies. In the present series, there were no death records following the procedure, although two cases required an emergent tracheotomy due to massive bleeding. Interestingly, both patients were treated for OSA by a multilevel procedure. There were 18 cases (4.3%) of major bleeding among a total of 47 bleeding cases (11.3%).

In the literature, the incidence of post-TORS bleeding varies widely, with reported rates ranging from 18.5% to 4.3% [8,9,20]. Focusing on the etiology of major bleeding, the reported rates range from 2% to 7% for patients treated for OSA (obstructive sleep apnea) and for oncological pathology, respectively [6,21,22,23,24]. Furthermore, in the literature, bleeding represents the most prevalent cause (approximately 30% of cases) of unplanned hospital readmission following TORS [25,26,27]. In the presented case series, bleeding occurred on average between the fourth and fifth postoperative day, while in the literature, bleeding typically occurs around the eighth to ninth postoperative day [9].

Furthermore, a proper management of postoperative pain is an important goal in reducing postoperative discomfort and stress for the patient, which could potentially lead to complications [28]. At our center, pain relief is provided by a combination of paracetamol and opioids. The dosage and mode of administration are determined case-by-case by a multidisciplinary team (surgeon and anesthetist), considering the patient and the procedure performed.

Intraoperative tracheotomy is a topic of major debate in the literature; in the present case series, it was performed in 324 (78.3%) patients with a mean decannulation time of 5.1 (SD ± 7.5) days. A comparative analysis of the two subgroups reveals that patients treated by TORS for OSA underwent tracheotomy more often that oncology patients (*p* < 0.001) but were decannulated earlier (*p* < 0.001). The high number of tracheotomies performed in OSA patients in the present series is determined by several factors. Firstly, most patients treated for OSA underwent a multistep procedure (75.6%) with concomitant surgery at the level of the palate and/or nasal septum. In such cases, we considered it appropriate to perform tracheotomy since it acts on several levels of the same upper airway. A further advantage of tracheotomy is that is allows patients to be transferred back to ward after surgery. They can also start feeding as early as the first day, thus avoiding nasogastric tube placement. However, different postoperative approaches to OSA patients have been described in the literature. Friedman et al. [29] rarely perform tracheotomy with extubation on the first day. In their opinion, this procedure can lead to difficult airway management in the event of a complication. Lin et al. [30] support the prolonged use of tracheal intubation, with the patient undergoing extubation the following day. It is recommended that a multidisciplinary team, including an anesthesiologist, should evaluate the airway management of OSA patients. The decision regarding the optimal approach should be made while also considering the available technologies and resources on site.

A salient finding of our study is that perioperative antiplatelet treatment is statistically associated with a five-fold increased risk of postoperative bleeding (*p* = 0.002, OR 4.59). Furthermore, the presence of a coagulation-disrupting disease was identified as a risk factor for postoperative bleeding (*p* = 0.049, OR 2.85). Also, the administration of anticoagulant therapy was identified as a risk factor for bleeding, a finding similar to that reported by Sharbel et al. [31] in a recent meta-analysis including 2008 patients. To date, in the literature, the role of anticoagulants is uncertain, with some studies identifying them as a risk factor and others finding them difficult to evaluate [27,32]. Consequently, considering the findings of our study, it is recommended to suspend antiplatelet therapy for patients scheduled for TORS. Whenever this is not feasible due to elevated cardiac risk, a closer follow-up and the eventual administration of tranexamic acid or other antihemorrhagic therapies may be considered. A multidisciplinary management (i.e., by a surgeon and an anesthesiologist) of these patients is crucial for assessing the patient and tailoring the treatment [33]. In our opinion, further studies will be necessary to assess the role of anticoagulant therapy as a risk factor for post-TORS bleeding and its potential perioperative management.

In the recent years, studies in the literature about the role of TORS in oropharyngeal carcinoma have increased. A number of clinical trials have been performed in order to assess the features of TORS as a first-line therapy and as a de-intensification strategy in the treatment of oropharyngeal carcinoma. TORS is typically recommended for small and advanced tumors [34,35,36,37]. In the oncological series included in this study, 12 cases of postoperative hemorrhage (11.3%) were reported, of which 7 (6.6%) were severe. Of these, eight patients received preoperative radiotherapy, and in the literature, preoperative radiotherapy has been indicated as a risk factor for bleeding [23].

Inconsistent results have been reported in the literature about the role of T, with some studies indicating that cT1-T2 patients exhibit a reduced risk of bleeding when compared to cT3-T4 patients [31]. However, these data were not confirmed by Kubik et al. [23] nor by our results, where T does not appear to be a risk factor for bleeding.

Since 2013, several studies have identified prophylactic AL during neck dissection as a protective factor for major bleeding [9,23,24,38,39]. The hypothesis that AL can prevent postoperative bleeding has been proposed, as per the ECOG 3311 study (NCT01898494), which required that all treated patients undergo prophylactic major vessel ligation during TORS [40,41]. Potential complications of this procedure include poor wound healing, fistula, impaired response to RT, and first-bite syndrome [42]. In the present case series, AL was performed in 24 cases (22.6%) during neck dissection, and no complications were observed to be caused by this procedure. The findings of this study show that this surgical intervention does not effectively mitigate overall postoperative bleeding; however, it does statistically significantly reduce the occurrence of major bleeding.

OSA is a health problem that is often underestimated, with significant social and economic implications. Since TORS was introduced as an effective technique for tongue base treatment, numerous authors have achieved satisfactory results. TORS is widely regarded as the gold-standard procedure for treating the base of the tongue, when it is available [3,21,43,44]. A recent meta-analysis by Miller et al. [45] has shown that tongue base reduction in TORS results in a significant reduction in AHI and in an improvement in daytime sleepiness and snoring in patients who have failed first-line therapy. The meta-analysis also demonstrates that bleeding is the most prevalent postoperative complication.

The present study also reveals that neither the severity of OSA nor multilevel surgical interventions, such as TORS accompanied by pharyngoplasty and/or septoplasty, are associated with an increased risk of postoperative bleeding. Therefore, multilevel treatment is a safe therapeutic option for postoperative bleeding and is comparable to isolated tongue base treatments. Upper airway collapse is frequently multilevel, with the retropalatal and retrolingual sites being the most affected; a simultaneous obstruction of these two levels has been observed in 25–33% of cases [46]. Treatment failure after single-level surgery is often determined by the presence of an untreated secondary collapse site. Therefore, it is imperative to carefully assess the collapse sites using Drug-Induced Sleep Endoscopy (DISE) before treating OSA patients. Recent studies have shown that surgical planning based on the Muller maneuver alone changes in 40–50% of patients after DISE [21,46,47]. Multilevel surgery has reported as the optimal treatment modality for a considerable proportion of OSA patients, even within a single-stage approach; recent studies have demonstrated the efficacy of the combination (TORS + pharyngoplasty) in the treatment of moderate–severe OSA [21,46,47].

Finally, Iannella et al. [48] demonstrated that multilevel treatment and continuous positive airway pressure (CPAP) have a comparable positive impact on the patient’s quality of life. Thus, it is likely that, when multilevel treatment is indicated in a single intervention, TORS is a safe and effective procedure that does not increase the risk of postoperative bleeding.

The major drawbacks of this study are (i) the fact that it was a single-center trial and (ii) its retrospective nature.

## 5. Conclusions

TORS is a surgical technique frequently performed for the management of head and neck disease, and bleeding is a major complication associated with this procedure. According to the present findings, patients treated with perioperative antiplatelets have an almost fivefold increased risk of developing postoperative bleeding, while the pathology (oncology vs. OSA) is not a risk factor for bleeding. Furthermore, according to our results, prophylactic artery ligation can significantly lower the risk of major bleeding, whereas in the OSA subgroup, multilevel surgery does not increase the risk of bleeding.

In our opinion, the identification of risk factors that may predispose to bleeding is a key step in the management of these patients, particularly from the perspective of a personalized approach. Further multicenter and prospective studies will be necessary to confirm these results and to obtain a further reliable risk assessment.

## Figures and Tables

**Table 1:** Clinical and pathological features of the studied population.

		**Cohort (** ***n*** ** = 414)**
Disease		
	OSA	308 (74.5%)
	Oncologic	106 (25.5%)
Gender		
	Male	323 (78%)
	Female	91 (22%)
Average Age at Surgery (±SD)		54.09 (±12.45)
Smoking		
	Yes	84 (32.7%)
	No	173 (67.3%)
Alcohol		
	Yes	136 (54.8%)
	No	112 (45.2%)
BMI		
	Normal weight	76 (20.9%)
	Overweight	187 (51.5%)
	Obese	100 (27.6%)
Hypertension		
	Yes	123 (49.2%)
	No	127 (50.8%)
Diabetes Mellitus		
	Yes	21 (8.9%)
	No	214 (91.1%)
Cardiac Disease		
	Yes	30 (12.8%)
	No	205 (87.2%)
Pulmonary Disease		
	Yes	19 (7.7%)
	No	229 (92.3%)
Coagulopathy		
	Yes	9 (3.6%)
	No	239 (96.4%)
Renal Disease		
	Yes	11 (4.4%)
	No	237 (95.6%)
Liver Disease		
	Yes	8 (3.2%)
	No	240 (96.8%)
Reumathologic Disease		
	Yes	12 (4.8%)
	No	236 (95.2%)
Thyroid Disease		
	Yes	18 (7.6%)
	No	218 (92.4%)
Gastrointestinal Diseases		
	Yes	15 (6.4%)
	No	221 (93.6%)
GERD		
	Yes	44 (18.6%)
	No	192 (81.4%)
Hypercholesterolemia		
	Yes	18 (7.6%)
	No	218 (92.4%)
Previous Cancer		
	Yes	18 (7.6%)
	No	218 (92.4%)
Genetic Disorders		
	Yes	9 (3.7%)
	No	235 (96.3%)
ASA		
	1	23 (8.7%)
	2	194 (73.2%)
	3	45 (17%)
	4	3 (1.1%)
STOP BANG		
	High	132 (71%)
	Low	54 (29%)
El Ganzouri		
	Probable difficult orotracheal intubation	46 (24.7%)
	Not probable difficult orotracheal intubation	140 (75.3%)
Anticoagulant		
	Yes	86 (21.5%)
	No	314 (78.5%)
Antiplatelet		
	Yes	18 (4.5%)
	No	382 (95.5%)
INR		
	Normal	378 (95%)
	High	20 (5%)
APTT		
	Normal	136 (54.8%)
	High	112 (45.2%)
Platelet count		
	Normal	380 (94%)
	High	22 (6%)
Intraoperative Tracheotomy		
	Yes	324 (78.3%)
	No	80 (21.7%)
Average Decannulation Time (±SD) (days)		5.1 (±7.5)
Bleeding		
	Yes	47 (11.3%)
	No	367 (88.7%)
Major Bleeding		
	Yes	18 (4.3%)
	No	396 (95.7%)
Average Postoperative Bleeding Time (±SD) (Days)		4.51 (±3.6)

Abbreviations: SD: standard deviation; OSA: obstructive sleep apnea; BMI: body mass index; GERD: gastroesophageal reflux disease; INR: International Normalized Ratio; APTT: activated partial thromboplastin time; ASA: American Society of Anesthesiology Classification.

**Table 2:** Clinical and pathological features of the studied population and association with bleeding.

		No Bleeding	Bleeding	*p*-Value
Hypertension	No	112	13	0.692
	Yes	108	15	
Diabetes	No	189	23	0.873
	Yes	18	3	
Cardiological Disease	No	182	21	0.354
	Yes	24	6	
Pneumological Disease	No	204	23	0.434
	Yes	16	3	
Coagulopathy	No	214	23	0.057
	Yes	6	3	
Renal Diseases	No	211	24	0.327
	Yes	9	2	
Hepatic Diseases	No	215	23	0.041
	Yes	5	3	
Rheumatologic Diseases	No	209	25	1.000
	Yes	11	1	
Thyroid Diseases	No	191	25	0.725
	Yes	17	1	
Gastrointestinal Diseases	No	196	23	0.224
	Yes	12	3	
GERD	No	170	20	0.595
	Yes	38	6	
Hypercholesterolemia	No	191	25	0.701
	Yes	17	1	
Previous Cancer	No	191	25	0.701
	Yes	17	1	
Genetic Disorders	No	210	23	0.235
	Yes	7	2	

Abbreviation: GERD: gastroesophageal reflux disease.

**Table 3:** Multivariate logistic regression analysis of bleeding.

	Bleeding	
Risk Factors	OR (IC 95%)	*p*-Value
Pathology (Oncologic vs. OSA)	0.43 (0.04–4.12)	0.466
Age	0.64 (0.07–5.64)	0.682
Smoking	0.93 (0.75–2.63)	0.279
Alcohol	0.17 (0.14–7.32)	0.987
BMI	0.12 (0.33–1.37)	0.850
Hypertension	0.57 (0.10–3.29)	0.528
Diabetes	0.68 (0.04–2.45)	0.790
Cardiovascular Diseases	0.33 (0.03–1.47)	0.401
Coagulopathy	2.85 (0.13–5.70)	0.049
Anticoagulant	0.96 (0.80–2.73)	0.286
Antiplatelet	4.59 (1.71–7.47)	0.002
Platelet count	0.74 (0.04–4.39)	0.576
ASA	0.83 (0.16–1.48)	0.822
STOP BANG	3.05 (0.52–6.62)	0.094
Gastrointestinal Disorders	0.65 (0.05–4.39)	0.732
GERD	1.23 (0.23–3.88)	0.359

Abbreviation: OR: odds ratio; OSA: obstructive sleep apnea; BMI: body mass index; GERD: gastroesophageal reflux disease; ASA: American Society of Anesthesiology Classification.

**Table 4:** Multivariate logistic regression analysis of severe bleeding.

	Major Bleeding	
Risk Factors	OR (IC 95%)	*p*-Value
Disease (oncologic vs. OSA)	1.28 (0.04–4.87)	0.484
Smoking	1.69 (0.04–4.69)	0.272
Alcohol	1.18 (0.14–2.03)	0.292
BMI	1.27 (0.32–3.9)	0.461
Hypertension	1.55 (0.97–6.56)	0.544
Coagulopathy	1.69 (0.89–1.87)	0.353
Anticoagulant	1.51 (0.32–4.39)	0.306
Antiplatelet	2.02 (0.49–7.67)	0.385

Abbreviation: OR: odds ratio; OSA: obstructive sleep apnea; BMI: body mass index.

**Table 5:** Comparison between the OSA group and the oncology group.

	Oncologic TORS	OSA TORS	*p*-Value
M/F ratio	3.3/1	3.8/1	0.191
Average age (±SD) (years)	65 (±9.6)	50.34 ± 11.02	<0.001
Number of intraoperative trachotomies (%)	56 (52.8%)	268 (87%)	<0.001
Average decannulation time (±SD) (days)	9.9 (±17)	4.2 ± 2.08	<0.001

Abbreviation: SD: standard deviation; OSA: obstructive sleep apnea; M: male; F: female.

**Table 6:** Clinical and pathological features of the oncological population.

		Cohort (*n* = 106)
Gender		
	Male	79 (74.5%)
	Female	24 (25.5%)
Average age at surgery (±SD)		65 (±9.6)
Site		
	Palatine tonsil	45 (42.6%)
	BoT	42 (39.7%)
	Supraglottic	6 (5.6%)
	Unknown primary	4 (3.7%)
	Soft palate	3 (2.8%)
	Posterior wall of the pharynx	3 (2.8%)
	Hypopharynx	3 (2.8%)
cT		
	1	56 (54.9%)
	2	35 (34.3%)
	3	11 (10.8%)
cN		
	0	47 (44.3%)
	1	27 (25.5%)
	2a	13 (12.3%)
	2b	14 (13.2%)
	2c	3 (2.8%)
	3	2 (1.9%)
Preoperative RT		
	Yes	8 (7.5%)
	No	98 (92.5%)
Neck dissection		
	Yes	86 (81.1%)
	No	20 (18.9%)
Arterial ligation		
	Yes	24 (22.6%)
	External carotid artery	15
	Lingual artery	6
	No	82 (77.4%)
Grading		
	G1	6 (8.6%)
	G2	23 (32.9%)
	G3	41 (58.5%)
p16		
	Positive	54 (50.1%)
	Negative	52 (49.9%)
Intraoperative tracheotomy		
	Yes	56 (52.8%)
	No	50 (47.2%)
Average decannulation time (±SD) (Days)		9.9 (±17)
Postoperative bleeding		12 (11.3%)
Postoperative major bleeding		7 (6.6%)
Average postoperative bleeding time (±SD) (days)		4.09 (±4)

Abbreviation: SD: standard deviation; BoT: base of tongue; RT: radiotherapy.

**Table 7:** Multivariate logistic regression analysis of bleeding in the oncological population.

	Bleeding	
Risk Factor	OR (IC 95%)	*p*-Value
P16	0.32 (0.04–0.83)	0.256
cT	0.61 (0.27–1.7)	0.436
cN	1.12 (0.49–2.56)	0.785
Site	1.35 (0.65–2.52)	0.463
Grading	0.47 (0.01–2.35)	0.370
Charlson Comorbidity Index	0.84 (0.46–1.51)	0.556
Neck Dissection	1.58 (0.01–6.23)	0.362

Abbreviation: OR: odds ratio.

**Table 8:** Analysis of major bleeding in the oncological population.

		Major Bleeding No	Major Bleeding Yes	*p*-Value
Preoperative RT	No	87	11	0.631
	Yes	7	1	
Arterial Ligation	No	53	6	<0.001
	Yes	23	1	

Abbreviation: RT: radiotherapy.

**Table 9:** Clinical and pathological features of the OSA population.

		Cohort (*n* = 308)
Gender		
	Male	244 (79.2%)
	Female	64 (20.8%)
Average age at surgery (±SD)		50.34 (±11.02)
Average preoperative AHI (±SD)		39.68 (±20.8)
Average preoperative LOS (±SD)		77.3 (±9.9)
OSA severity		
	Mild	30 (10.9%)
	Moderate	75 (27.4%)
	Severe	169 (61.7%)
Other surgical procedures		
	Septoplasty	83 (26.9%)
	Pharyngoplasty	216 (70.1%)
	Septoplasty + Pharyngoplasty	233 (75.6%)
Intraoperative tracheotomy		
	Yes	268 (87%)
	No	40 (13%)
Average decannulation time (±SD) (days)		4.2 (±2.08)
Postoperative bleeding		35 (11.4%)
Postoperative major bleeding		11 (3.6%)
Average postoperative bleeding time (±SD) (days)		4.77 (±3.7)

Abbreviation: SD: standard deviation; OSA: obstructive sleep apnea; AHI: Apnea Hypopnea Index; LOS: Lowest Oxygen Saturation.

**Table 10:** Multivariate logistic regression analysis of bleeding in the OSA population.

	Bleeding	
Risk Factor	OR (IC 95%)	*p*-Value
OSA Severity	1.58 (0.78–2.97)	0.214
Other Surgical Procedures	0.04 (0.01–30.91)	0.993
Pharyngoplasty	14.28 (0.01–31.20)	0.993
Septoplasty	1.36 (0.98–2.28)	0.443

Abbreviation: OR: odds ratio; OSA: obstructive sleep apnea.

## Data Availability

The original contributions presented in this study are included in the article. Further inquiries can be directed to the corresponding author.
